# Acquisition of a gene cluster in *Clostridioides difficile* PCR ribotype 023 strains enables xylitol utilization

**DOI:** 10.1128/msphere.00263-26

**Published:** 2026-07-16

**Authors:** Katherine J. Wozniak, Lei Pan, Duolong Zhu, Jeroen Corver, Ed J. Kuijper, Wiep Klaas Smits, Robert A. Britton

**Affiliations:** 1 Department of Molecular Virology & Microbiology, Baylor College of Medicine 189531, Houston, Texas, USA; 2 Leiden University Center for Infectious Diseases (LUCID), Leiden Medical Center 4501, Leiden, the Netherlands; 3 Alkek Center for Metagenomics & Microbiome Research, Baylor College of Medicine 661296, Houston, Texas, USA; The University of Iowa, Iowa City, Iowa, USA

**Keywords:** *Clostridium difficile*, xylitol, RT023, xdh, mobile genetic elements

## Abstract

Hundreds of ribotypes of the gastrointestinal pathogen *Clostridioides difficile* have emerged over the last three decades, yet the factors driving their emergence are poorly understood. Recently, there has been an increase in infections caused by PCR ribotype 023 (RT023) strains in Europe. We profiled the growth of seven RT023 strains in 190 unique carbon sources and found they were able to grow in xylitol, a sugar alcohol used as a food additive in humans and animals. Other ribotypes of *C. difficil*e tested (*n* = 19) displayed little to no growth in 0.5% xylitol and were growth-inhibited in higher concentrations of xylitol. Genome sequencing identified that RT023 strains acquired a putative xylitol dehydrogenase (*xdh*) gene in a mobile genetic element (MGE) that is absent from other *C. difficile* ribotypes. We created a deletion of *xdh* in the RT023 strain PRB1128 and observed poor growth in xylitol, indicating that the *xdh* is necessary for xylitol utilization. Complementation of the *xdh* mutant with a plasmid-based inducible copy of the *xdh* gene restored growth in xylitol. We performed competition assays in minibioreactor arrays (MBRAs) and observed that PRB1128 outcompeted the non-xylitol-utilizing strain CD2015 (RT027) in the presence of xylitol. These data support that the *xdh* gene within RT023 strains provides a fitness benefit for growth in xylitol. Interestingly, the chromosomal locus where the MGE inserted appears to be a hotspot for genetic insertions across clades of *C. difficile*. Together, this work improves our understanding of the molecular basis for niche adaptation of *C. difficile*.

**IMPORTANCE:**

Genetic factors aiding in the emergence of the opportunistic pathogen *Clostridioides difficile* are poorly understood. Infections with clade 3 (PCR ribotype 023) strains causing severe disease have increased since 2008. Here, we show RT023 strains have the unique ability to utilize xylitol, a sugar alcohol used as a food additive in humans and animals, due to the presence of a xylitol dehydrogenase (*xdh*) gene within a mobile genetic element (MGE). This xylitol utilization ability confers a fitness benefit in competition against other *C. difficile* ribotypes, as well as in a fecal community *in vitro*. Research investigating the underlying genetic factors driving the physiology of *C. difficile* will improve our understanding of colonization and hypervirulence.

## INTRODUCTION

*Clostridioides difficile* is a gram-positive, toxin-producing anaerobe that is one of the most common hospital-associated gastrointestinal infections and contributes to an estimated $5 billion annual cost to the healthcare system in the United States (1–3). *C. difficile* causes a range of gastrointestinal symptoms including abdominal pain and diarrhea, and in severe cases, pseudomembranous colitis and death (3). Infections with *C. difficile* have risen since the year 2000, likely driven by antibiotic use that is disruptive to the endogenous gut microbiome (4). Pathogenic *C. difficile* is generally organized into five phylogenetic clades based on whole-genome sequence analyses (5). Specific ribotypes within clades have emerged in hospitals and exhibit severe disease, with significantly higher morbidity and mortality than other strains (6–8). *C. difficile* pathogenicity is multifactorial, where toxin production, sporulation efficiency, and antibiotic resistance are contributors (9, 10). Further, unique carbon utilization ability and heterogeneous motility have also been linked to high levels of toxin production (11–14).

Since 2008, clade 3 strains (dominated by PCR ribotype RT023) (15) have emerged in hospitals in the United Kingdom and across Europe, exhibiting severe disease (8). RT023 strains have a pathogenicity locus (PaLoc) that contains a mobile element (Tn*6218*) between *tcdE* and *tcdA* (16), and encodes binary toxin (8), and a four-gene trehalose cluster (8) similar to that in RT078 (11). RT023 strains have glycosylated S-layer proteins, which have been shown to aid in adherence to Caco2 cells (15), a truncated gene encoding the protease PPEP-1 which could hinder lifestyle switching (15), and are highly motile *in vitro* (17). Finally, a survey of emerging *C. difficile* ribotypes showed that RT023 strains grew more poorly than strains from other clades in a variety of carbon sources, including glucose (18), and are unable to hydrolyze esculin, the glucoside in commonly used chromogenic agar (19).

Xylitol is a five-carbon sugar alcohol found naturally in small amounts in fruits and vegetables (20). Recently, xylitol has become a more common sweetener in confectioneries and baking because it is less caloric than sucrose but has a comparable sweet taste (20). The human body itself produces 5–15 g of xylitol a day through glucose metabolism, where most of the production occurs in the liver (21). Fifty percent of ingested xylitol is absorbed into the bloodstream through passive diffusion in the small intestine (21). The other 50% of consumed xylitol enters the colon, where some members of the colonic microbiome can metabolize it to make short-chain fatty acids (22). Several studies have found that xylitol consumption changes the microbiome by increasing the amount of phylum *Bacillota* (formerly *Firmicutes*) and genus *Prevotella*, while decreasing phylum *Bacteroides* in the gut microbiome (23). The remaining undigested xylitol can increase water retention and have a laxative effect (24). Xylitol dehydrogenase is the enzyme responsible for converting xylitol into xylulose, which is processed further by the pentose phosphate pathway, glycolysis, and amino acid synthesis (25). Xylitol dehydrogenase is commonly found in yeast but not in bacteria (20). For most bacteria, xylitol is toxic as they cannot metabolize xylitol, and its transport and export impose an energy cost (24). As such, chewing gum frequently contains xylitol to prevent dental caries (26).

We sought to understand the underlying molecular features of RT023 strains that might contribute to their fitness. Here, we report that RT023s uniquely metabolize xylitol due to the presence of a putative xylitol dehydrogenase (*xdh*) gene within a mobile genetic element (MGE). Further, *xdh* was necessary to confer a growth benefit in xylitol, and complementation of *xdh* restores growth. Presence of *xdh* conferred a fitness advantage when competing against non-xylitol-utilizing *C. difficile* in the presence of xylitol. Additionally, an RT023 strain (PRB1128) stably colonized an antibiotic-treated microbiome *in vitro* in both the presence and absence of xylitol. We conclude that RT023 evolved the ability to utilize xylitol through the acquisition of a mobile genetic element, perhaps from two separate recombination events from *Blautia producta* or *Enterococcus faecalis*. The locus of this MGE appears to be a hotspot for genetic diversity across clades of *C. difficile*, and the underlying selection for these genes to be maintained remains unstudied.

## RESULTS

### RT023 strains grow in xylitol, while xylitol is detrimental to growth of non-RT023s

**Fig 1:**
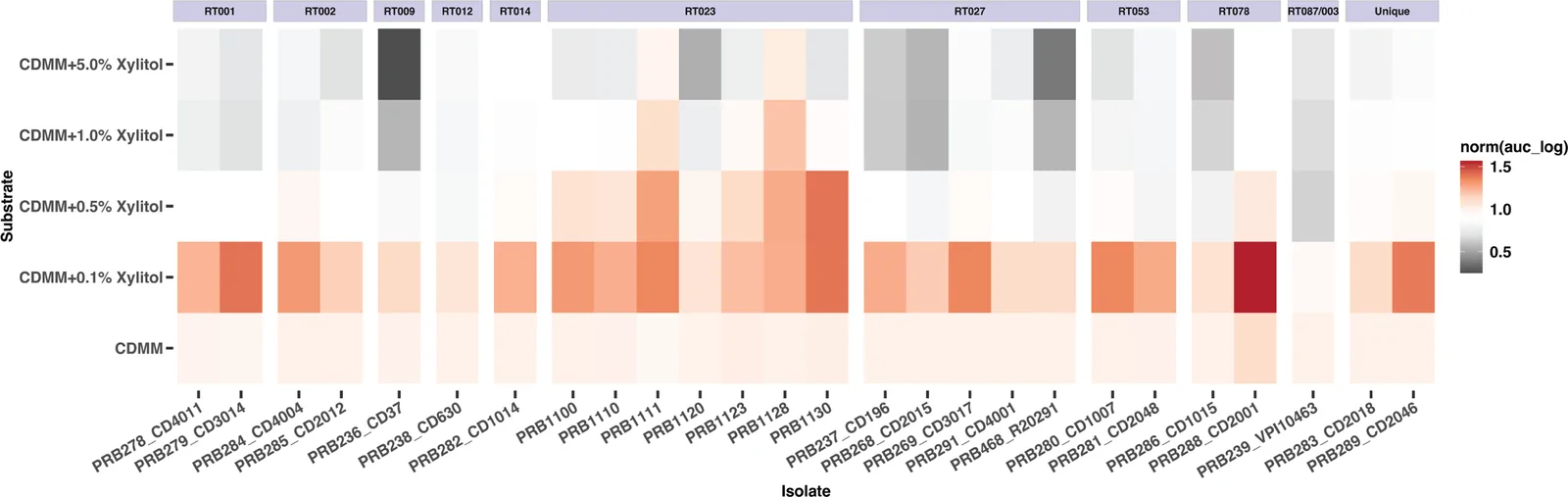
RT023 strains uniquely metabolize xylitol. Normalized carrying capacity, as measured by area under the curve (AUC)*,* of different clades of *C. difficile* grown in CDMM with xylitol up to 5%. Log-transformed AUC displays the total growth supported by the medium over time. Carbon-supportive growth is indicated by red tiles (AUC > 1.0), neutral impact is indicated by white tiles (AUC = 1.0), and growth inhibition is indicated by gray tiles (AUC < 1.0). Log-transformed AUCs of RT023 strains are significantly different from all non-RT023s in 0.5% xylitol (Wilcoxon rank-sum test, *P* < 2.2E−16).

To assess carbon metabolic profiles of *Clostridioides difficile*, we performed Biolog analyses of 26 strains spanning the five clades in chemically defined minimal media (CDMM) (18), supplemented with single carbon sources. We initially observed that strains of RT023s were able to grow in xylitol, while strains from other clades stopped growing or lost density in xylitol. A previous study showed RT023 strains grew poorly in most simple sugars compared to emerging ribotypes from other clades (18). Based on this observation, we assessed the effect of xylitol on the growth of *C. difficile* in more detail, with a focus on RT023. We confirmed that seven strains of RT023 were able to grow in CDMM supplemented with 0.5% xylitol, and that two of the seven strains able to grow in up to 5% xylitol (Fig. 1). We observed some growth in 0.5% xylitol in the non-RT023 strains CD4004 (RT002), CD1014 (RT014), and CD2001 (RT078), and two unique strains (CD2018 and CD2046), but not to the level observed in RT023 strains (Fig. 1).

### RT023 strains acquired a putative xylitol dehydrogenase within a mobile genetic element (MGE)

**Fig 2:**
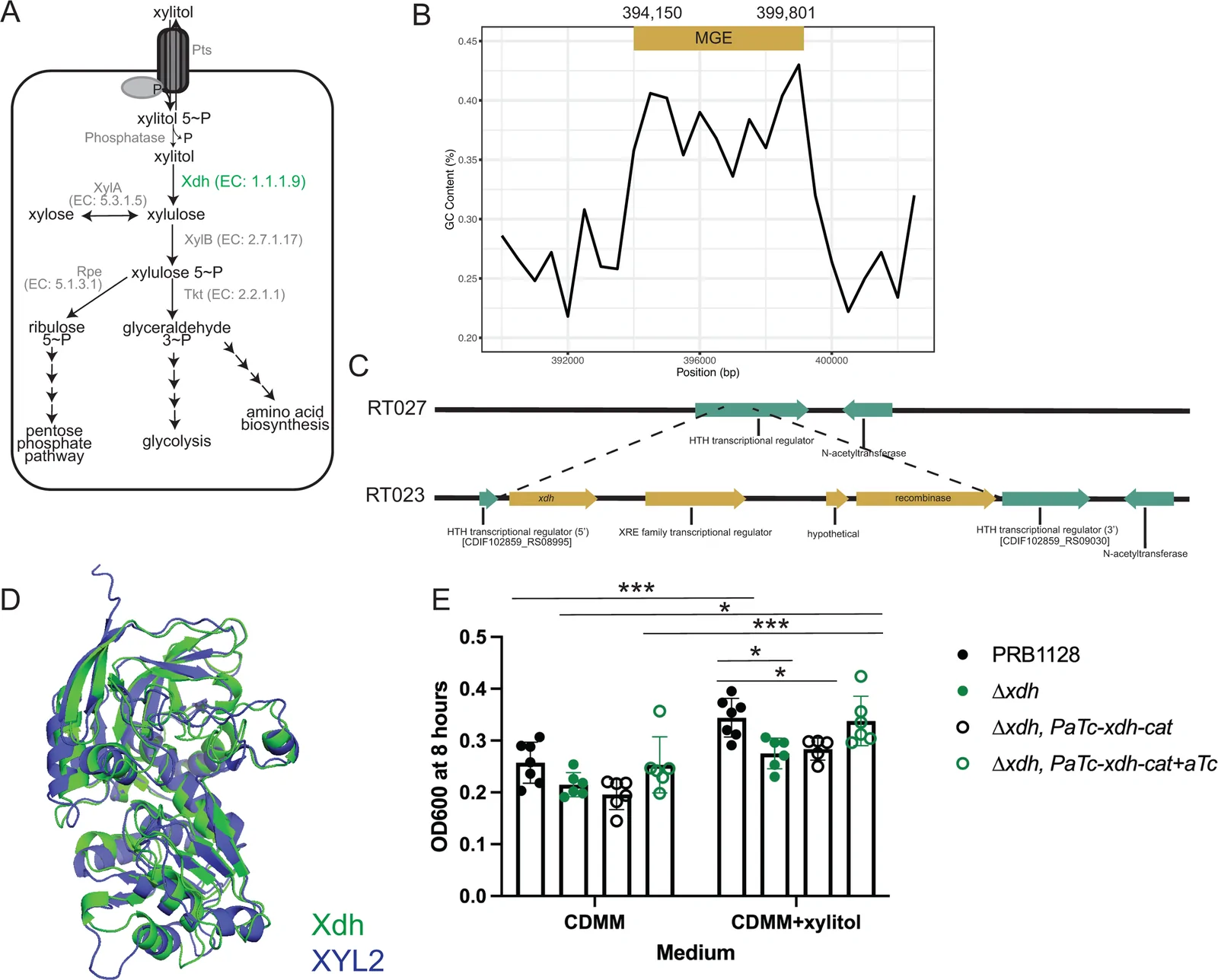
Identification of a putative xylitol dehydrogenase in RT023s. (**A**) Abbreviated xylitol utilization pathway necessary to catalyze major steps with protein names from *C. difficile* and Enzyme Commission (EC) numbers. Xylitol dehydrogenase written in green (EC: 1.1.1.9). (**B**) GC content in the MGE neighborhood in RT023 strains. (**C**) Genomic schematic of mobile genetic element (MGE) insertion site (top) in the RT027 genome. In R20291, this site corresponds to the transcriptional regulator locus: WP_018112778. The MGE containing *xdh* in RT023 strains is shown on the bottom with endogenous genes in teal, MGE genes in gold. Genomic position of the transcriptional regulator pieces in PRB1128 (RT023) indicated below gene annotation. (**D**) Structural prediction of Xdh aligned to XYL2 (yeast) generated from AlphaFold (RMSD = 1.798 Å). (**E**) Optical density at 8 h of PRB1128, ∆*xdh*, and the complement strain (∆*xdh*, *PaTc-xdh-cat*) in CDMM ±0.5% xylitol and ±anhydrotetracycline (aTc) induction, where applicable. *n* = 6–8 biological replicates per group. Means with standard deviation plotted. A two-way ANOVA and Tukey post hoc tests were performed. PRB1128 in CDMM vs CDMM + xylitol (***P < 0.0001). ∆*xdh* in CDMM vs. CDMM + xylitol (*P < 0.05). PRB1128 in CDMM + xylitol vs ∆*xdh* in CDMM + xylitol (*P < 0.05). Complemented strain (∆*xdh*, *PaTc-xdh-cat + aTc*) in CDMM + xylitol vs PRB1128 in CDMM + xylitol (n.s.). ∆*xdh* in CDMM + xylitol vs ∆*xdh, PaTc-xdh-cat* + aTc in CDMM + xylitol (*P < 0.05). PRB1128 in CDMM + xylitol vs ∆*xdh*, *PaTc-xdh-cat* (without induction) in CDMM + xylitol (*P < 0.05). A complete list of pairwise *P* values can be found in Table S2.

We searched for the presence of a putative xylitol dehydrogenase (EC: 1.1.1.9) that would aid in xylitol metabolism of RT023 strains (Fig. 2A). We identified a single gene annotated as a threonine dehydrogenase that was unique to RT023s within an element of high-GC content (38% GC compared to 28% overall in the genome) (Fig. 2B), suggesting it was acquired horizontally. In addition to the putative xylitol dehydrogenase, the mobile genetic element (MGE) encodes an XRE family transcriptional regulator, a hypothetical protein, and a recombinase (Fig. 2C). We found that the mobile element integrated into a helix-turn-helix (HTH) transcriptional regulator gene, resulting in two gene fragments (CDIF102859_RS08995 and RS09030). Interestingly, this gene is intact in the RT027 strain R20291 (WP_018112778). Since *xdh* in *C. difficile* lacks significant sequence identity to canonical xylitol dehydrogenases, we created an AlphaFold prediction for structural comparison. The resulting model of this putative xylitol dehydrogenase is structurally similar to an AlphaFold model of XYL2 (UniProt: Q07993), a xylitol dehydrogenase in yeast that catalyzes the conversion of xylitol into xylulose (RMSD: 1.798 Å) (Fig. 2D). Importantly, the residues for xylitol specificity, cofactor binding, and zinc coordination are conserved at the appropriate sites in the structural model. Based on this information, as well as further characterization below, we will hereafter refer to the gene as *xdh* (for *x*ylitol *d*e*h*ydrogenase). We selected a strain (PRB1128) that grew in up to 5% xylitol in Biolog assays (Fig. 1). We grew PRB1128 in batch for 8 h in CDMM and observed that xylitol improved growth (Fig. 2E).

To determine whether the putative xylitol dehydrogenase (*xdh*) is required for xylitol metabolism, we generated a clean deletion using CRISPR/Cas12a (27) in PRB1128 (Fig. 2E). Using the ∆*xdh* strain, we observed a decrease in optical density compared to the parental strain in the presence of 0.5% xylitol (*P < 0.05) (Fig. 2E). We complemented ∆*xdh* using pRPF185-derived plasmid (28) (harboring a thiamphenicol resistance gene) driving *xdh* expression from an anhydrotetracycline-inducible promoter (∆*xdh*, *PaTc-xdh-cat*). Without induction of the *xdh* gene, the complement strain is not significantly different from ∆*xdh* in CDMM. Upon induction of the *xdh* gene in the presence of xylitol, we observed growth like that of WT grown in xylitol, suggesting restoration of the parental phenotype (Fig. 2E). Together, these data show *xdh* is necessary for xylitol utilization in PRB1128.

### The mobile genetic element (MGE) locus is a hotspot for genetic diversity across *C. difficile*

We next queried the genomic content at the MGE locus in other clades of *C. difficile* and observed MGEs inserted into the HTH transcriptional regulator at different positions (Fig. S1). Namely, we observed instances of distinct MGEs in RT010 (clade 1) and CD161 (clade 4) (Fig. S1). In RT010, we observed genes encoding a recombinase, hypothetical, relaxase, replication initiator, VLP-containing gene, ATPase, transcriptional regulator, N-acetyltransferase, topoisomerase (partial), peptidase, *ermB*, peptidase, topoisomerase (partial), *parA*, peptide-binding, antitoxin, zeta family toxin, and ATPase at this genomic locus. In CD161, we observed genes encoding two hypothetical proteins, *aadE*, N-acetyltransferase, transcriptional regulator, bifunctional aminoglycoside N-acetyltransferase AAC(6′), N-acetyltransferase, conjugal transfer, *repA*, plasmid recombinase, hypothetical, and DUF4368 containing gene at this locus. In addition, we observed conservation of the MGE of PRB1128 in other RT023 strains (DSM 102978 and DSM 102860) (7). In strains from clade 2, such as CD630 and R20291, we determined that the HTH transcriptional regulator gene remained intact (Fig. S1).

### The *xdh*-containing MGE was likely horizontally transferred from other gut bacteria

The high-GC content of the RT023-specific MGE suggests it may have been horizontally transferred. To investigate possible sources, we used BLAST to search for homologous genes in the NCBI nucleotide database. We first queried the MGE genes as individuals and found that the XRE family transcriptional regulator, hypothetical protein, and recombinase share 92–94% identity with *Enterococcus faecalis* and *Blautia producta*, suggesting they could have originated from these organisms (Table S1). Notably, significant alignment to *xdh* was not found in *E. faecalis* or *B. producta* at the nucleotide level. The *xdh* gene was found with 100% identity to *C. difficile* genomes DSM 102860, DSM 102859, DSM 102978, RSS13, 744, and 741. DSM strains were described as RT023s previously (7). When we queried *xdh* and excluded *C. difficile*, we only identified hits in three strains of *Clostridium beijerinckii* at 75% identity with 30% coverage in the 5′ region of the *xdh* coding sequence.

Next, we queried the entire MGE. We found 100% identity with 100% query coverage to *C. difficile* genomes listed above. The next highest identity hits were contained within *E. faecalis* (IDRL-7538) and *B. producta* (ATCC 27340 and DSM 2950) with >94% identity and 73% query coverage encompassing the genes downstream of *xdh* (Fig. S2). Given that the MGE aligns to the transcriptional regulator (*xre*) through the recombinase in *E. faecalis* and *B. producta*, we suggest that these genes were transferred together as one unit. Many other bacteria have an NAD(P)-dependent alcohol dehydrogenase (<80% identical at an amino acid level) that could potentially act as a xylitol dehydrogenase, but none were found in *E. faecalis* or *B. producta*. These data suggest RT023 acquired genetic material horizontally, perhaps with *xdh* insertion into the MGE secondarily, to improve fitness.

### PRB1128 has a competitive advantage against a non-RT023 *C. difficile* in continuous flow mini-bioreactor arrays

**Fig 3:**
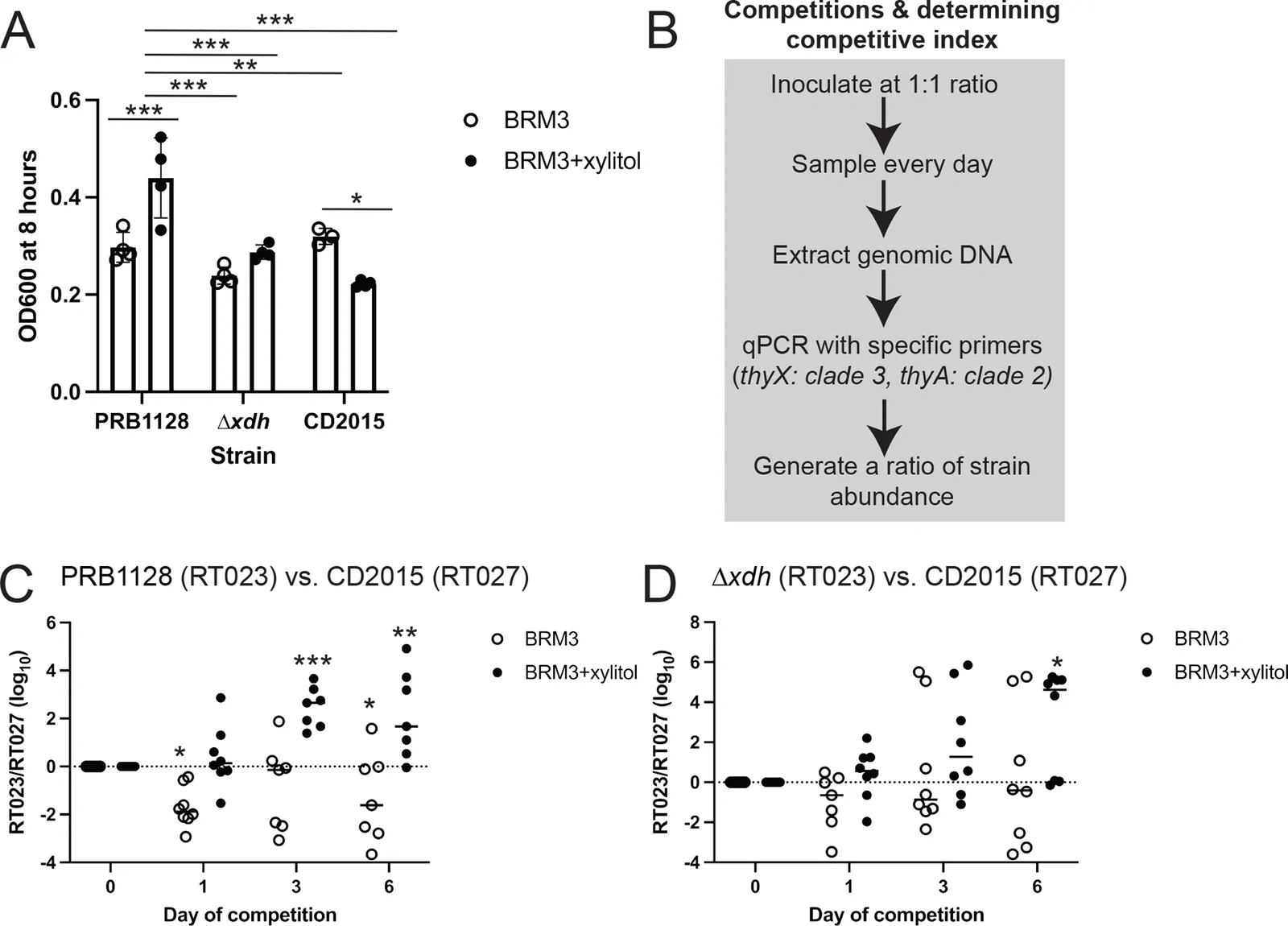
Steady-state bioreactor competitions of PRB1128 to determine competitive advantage in xylitol. (**A**) Optical density at 8 h of PRB1128 and ∆*xdh* in BRM3 ±0.5% xylitol. *n* = 3–4 biological replicates per group. Means with standard deviation plotted. A two-way ANOVA and Tukey’s multiple comparisons tests were performed. PRB1128 in BRM3 vs BRM3 + xylitol (***P < 0.0005). PRB1128 in BRM3 + xylitol vs ∆*xdh* in BRM3 + xylitol (**P < 0.005). CD2015 in BRM3 vs BRM3 + xylitol (*P < 0.05). PRB1128 in BRM3 + xylitol vs CD2015 in BRM3 + xylitol (***P < 0.0005). PRB1128 in BRM3 + xylitol vs ∆*xdh* in BRM3 and CD2015 in BRM3 (***P < 0.0005, **P < 0.005). (**B**) Schematic to determine competitive index from mini-bioreactor arrays. *thyX* is intact in RT023s and is therefore used to quantify RT023s. In non-RT023s, *thyX* is disrupted by *thyA* and several other genes. Therefore, *thyA* is used to quantify non-RT023s. (**C**) Competition assay between PRB1128 (RT023) and CD2015 (RT027). *n* = 7–8 biological replicates per group. Dashed line represents an equally matched pair. Bars represent the median. Competitive index is a ratio of RT023/RT027 (log_10_). The ratios of RT023 and RT027 were calculated using clade-specific qPCR primers (see Materials and Methods). A two-way ANOVA with Tukey post hoc tests was performed comparing all days of competition within the indicated medium. In the absence of xylitol, CD2015 is significantly more abundant than PRB1128 on day 1 (*P < 0.05) and day 6 (*P < 0.05). In the presence of xylitol, RT023 is significantly more abundant at days 3 and 6 than CD2015 (***P < 0.0005 and **P < 0.005, respectively). (**D**) Competition assay between **∆***xdh* (RT023) and CD2015 (RT027). *n* = 7–8 biological replicates per group. Dashed line represents an equally matched pair. Bars represent the median. Competitive index is a ratio of RT023/RT027 (log_10_). The ratios of RT023 (**∆***xdh*) and RT027 were calculated using clade-specific qPCR primers (see Materials and Methods). A two-way ANOVA with Tukey post hoc tests was performed comparing all days of competition within the indicated medium. In the absence of xylitol, ∆*xdh* (RT023) and CD2015 were equally matched on all days. In the presence of xylitol, strains are equally matched on all days except on day 6, when **∆***xdh* becomes more abundant than CD2015 (*P < 0.05).

We hypothesized the ability of RT023 strains to grow in xylitol would confer a competitive advantage over other hypervirulent *C. difficile* ribotypes. We assessed competitive advantage by competing strains against each other in continuous flow, steady state minibioreactor arrays (MBRAs). We first confirmed that PRB1128 still showed increased growth in bioreactor medium (BRM3) supplemented with xylitol. Similar to CDMM, we observed that PRB1128 increased in density in the presence of xylitol (Fig. 3A, ***P < 0.0005) while BRM3 supplemented with xylitol did not improve growth of ∆*xdh* (Fig. 3A). The hypervirulent CD2015 (RT027, clade 2) decreased in optical density in the presence of xylitol (Fig. 3A, *P < 0.05). Next, we performed *in vitro* competition assays using MBRAs (29) (Fig. 3B). We first competed PRB1128 against CD2015 in the presence and absence of 0.5% xylitol for 6 days. We observed that PRB1128 outcompeted CD2015 in the presence of xylitol at days 3 and 6 (Fig. 3C, closed circles, ***P < 0.0005, **P < 0.005). Conversely, CD2015 outcompeted PRB1128 in the absence of xylitol at days 1 and 6 (Fig. 3C, open circles, *P < 0.05). When cultures are grown in batch in BRM3 alone, CD2015 grows similarly to PRB1128 (Fig. 3A; Fig. S3A), while in xylitol, PRB1128 grows better than CD2015 (Fig. 3A; Fig. S3B). The growth rate of PRB1128 (*µ* = 0.215) compared to CD2015 (*µ* = 0.200) could perhaps explain the PRB1128 growth advantage in xylitol in MBRAs. To further confirm the necessity of *xdh* to improve fitness in xylitol, we competed ∆*xdh* against CD2015 in MBRAs (Fig. 3D). In BRM3 alone, ∆*xdh* is equally matched with CD2015 throughout the entire competition (Fig. 3D, open circles). In BRM3 + xylitol, strains were equally matched on days 1 and 3, but ∆*xdh* outcompeted CD2015 on day 6 (Fig. 3D, closed circles, *P < 0.05). ∆*xdh* grew to the lowest optical density in a batch BRM3 culture (Fig. 3A; Fig. S3A) but grew slightly better in xylitol than CD2015 (Fig. 3A; Fig. S3B). It is possible that other genomic features allow for some level of xylitol resistance in ∆*xdh* compared to CD2015, which could explain its maintained fitness on the last day of competition against a non-RT023 strain.

### PRB1128 can stably colonize an antibiotic-treated fecal community in minibioreactor arrays

**Fig 4:**
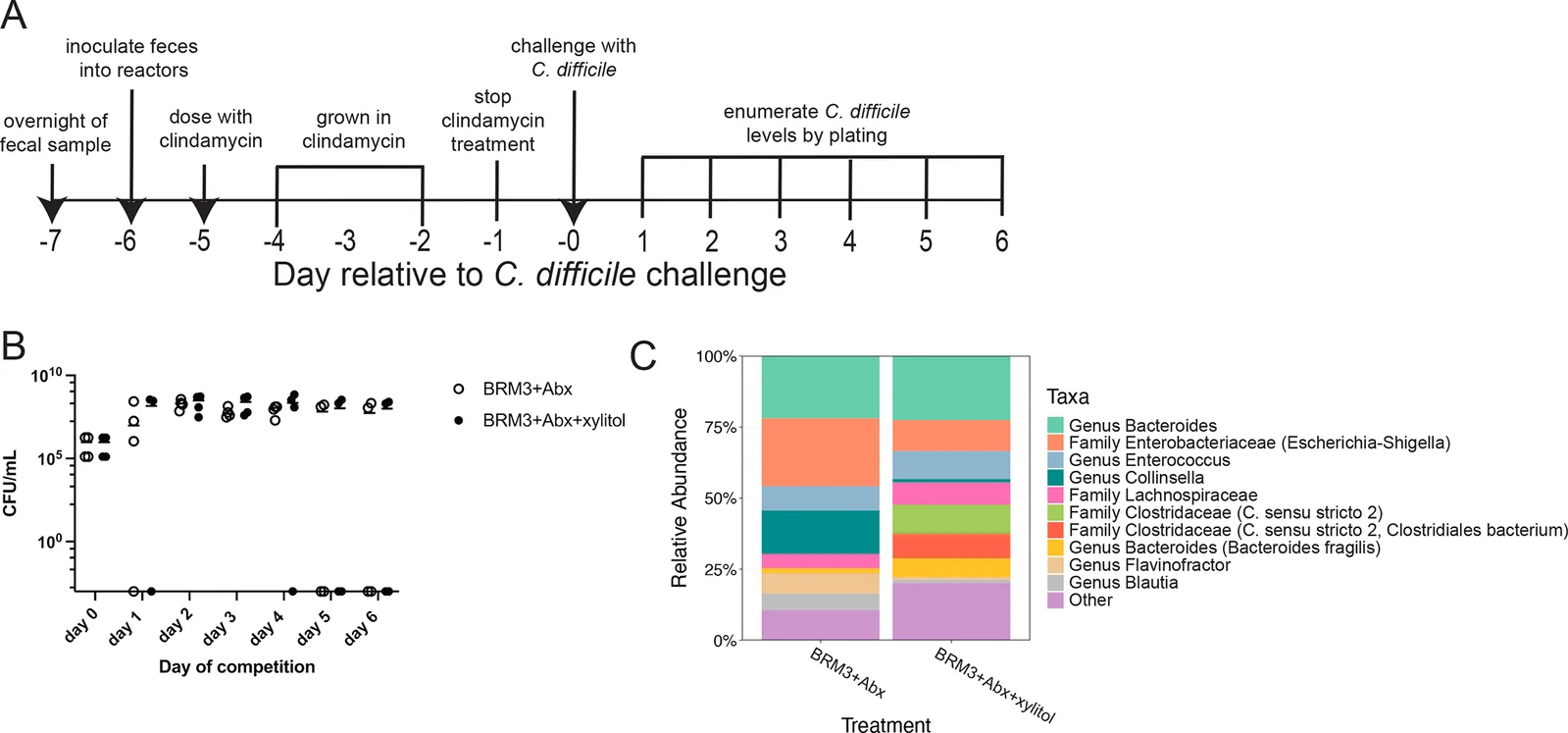
PRB1128 invasion of a fecal community in MBRAs. (**A**) Experimental scheme of *C. difficile* challenge in a microbiome. Days indicated are relative to inoculation with *C. difficile*. The community was either grown in BRM3 or BRM3 + 0.5% xylitol from day −6 (start) until day 6 (experimental end). (**B**) Quantification of PRB1128 (in CFU/mL) each day of competition in a fecal community. Bars represent the median. Steady, robust colonization in the presence of xylitol after 1 day, but no significant difference from BRM3 alone over the subsequent days. (**C**) Relative abundance on day 6 of competition of the top 10 taxa in fecal communities treated with clindamycin (BRM3 + Abx) or clindamycin and xylitol (BRM3xyl + Abx). In the presence of xylitol, other taxa increase in addition to *Bacteroides* and *Clostridiaceae*, while *Enterobacteriaceae*, *Collinsella, Blautia*, and *Flavonifractor* decrease.

We established a human fecal community in MBRAs and treated it with clindamycin (29) to mimic antibiotic disruption of the microbiome (Fig. 4A). We hypothesized that when a fecal community is challenged with PRB1128, the xylitol condition would allow for a greater capacity of colonization. We observed that PRB1128 stably colonized a fecal community in MBRAs over 6 days in the presence and absence of 0.5% xylitol to similar levels (Fig. 4B). To understand whether xylitol changes the community composition after antibiotic disruption, we performed 16S rRNA sequencing on the fecal community after clindamycin treatment and after clindamycin treatment in xylitol-containing medium at the end of the experiment (day 6). We observed that the presence of xylitol in addition to clindamycin shifted the relative abundances of the community (Fig. 4C). Specifically, we saw a decrease in *Enterobacteriaceae* and *Collinsella* and an increase in *Clostridiaceae* (Fig. 4C). This suggests the RT023 niche in an antibiotic-treated fecal community differs in the presence and absence of xylitol, and perhaps other *Clostridiaceae* can utilize xylitol, therefore limiting the carrying capacity of *C. difficile* PRB1128. Together, we conclude that the presence of *xdh* confers a fitness benefit to RT023 strains *in vitro*, but colonization of a fecal community is not dependent on xylitol presence.

## DISCUSSION

In this study, we uncovered a unique carbon utilization phenotype that sets clade 3 RT023 *C. difficile* strains apart from other strains tested. RT023 strains were able to utilize xylitol due to the presence of a xylitol dehydrogenase gene in a mobile genetic element (MGE). We deleted *xdh* and confirmed that it is necessary for xylitol utilization. Complementation using an inducible plasmid restores growth in xylitol-containing media. It appears that the *xdh* gene could have come from other *Clostridiaceae* while the transcriptional regulator, hypothetical gene, and recombinase likely arose from one transfer event from *B. producta* or *E. faecalis* based on nucleotide identity and coverage. We suggest this locus is a hotspot for genomic diversity across *C. difficile* clades, as we observed strains from clades 1 and 4 also have MGE inserted genes of different numbers and content. The ability to metabolize xylitol conferred a fitness advantage in bioreactors when competing against a non-RT023 strain. In addition, one RT023 strain that grows at high levels of xylitol (PRB1128) successfully colonized an antibiotic-treated fecal community in the presence and absence of xylitol to similar levels.

We cannot exclude the possibility that other RT023 strains behave differently from PRB1128, a strain selected because of the ability to grow well in high levels of xylitol. However, a 2020 study compared whole genomes of 86 clinical RT023 strains and showed that strains were >99.8% identical (8). This suggests RT023s are closely related and therefore we consider it more likely that the phenotypes we showed are similar in other RT023 strain backgrounds.

Despite increasing xylitol prevalence in our diet, it is not known whether the amount present is driving pathogen adaptation or virulence. Recently, there has been an increase in xylitol supplementation in livestock feed to aid in heat tolerance (30, 31). Separately, studies have demonstrated the presence of *C. difficile* in domestic and wild animals, including livestock (7). It is plausible that animal carriers are selecting for *C. difficile* strains due to diet, and these strains are consequently evolving higher virulence in humans, although no study has been done to surveil xylitol-fed livestock microbiomes for RT023 strains.

One study of xylitol-fed mice found that xylitol significantly enriched transcripts of xylitol dehydrogenase, xylulokinase, and xylulose phosphate isomerase in mouse colon microbiomes (23). They attributed the increase in xylitol dehydrogenase to the increase in *Flavonifractor*, *Clostridium*, *Providencia*, and *Lachnospiraceae* in the microbiome (23). Interestingly, no one bacterium harbored all enzymes for xylitol utilization (23). The authors hypothesized that xylitol dehydrogenase-containing bacteria produced xylose from xylitol and cross-fed the xylulokinase-containing bacteria (23). Further, they suggest that the resulting xylose enters the pentose phosphate pathway to aid in SCFA production (23). It is possible that xylitol-utilizing *C. difficile* strains are more successful because they metabolize xylitol entirely and therefore do not cross-feed the microbiome. In our own study, we observed that xylitol changed the community distribution of a clindamycin-treated fecal community *in vitro*. Specifically, *Enterobacteriaceae* and *Collinsella* decreased, while *Clostridiaceae* increased. We expected PRB1128 to colonize the microbiome to higher levels in BRM3 + xylitol, due to its competitive advantage in bioreactors, yet it colonized to similar levels as BRM3 alone. It is possible that xylitol restructures the fecal community composition, as xylitol can be bactericidal to some bacteria, while other bacteria have been shown to cooperate to degrade xylitol in mice (23). We suggest that the xylitol-driven bloom of other *Clostridiaceae* in the community reduced the carrying capacity of PRB1128 *C. difficile* in the community. Additionally, we hypothesize that xylitol would allow RT023 strains to outcompete non-RT023 strains for colonization of a fecal community. Additional studies are needed to determine the effects of xylitol on the fecal microbiome and the susceptibility of the microbiome for invasion by *C. difficile* RT023 strains.

It is possible that the mobile genetic element containing *xdh* is maintained in the chromosome due to its importance in xylitol utilization. Conversely, other genes contained within the MGE could have a regulatory role elsewhere in the genome, which contributes to maintenance. This MGE is not similar to other well-characterized MGEs and therefore cannot be classified. Because of this, it is unknown whether the element could excise and be taken up by other bacteria, causing a growth defect and a persister-like state for cells containing the MGE in the absence of xylitol (32). We found instances of other MGEs inserted in the same genomic locus in clades 1 and 4, suggesting this site is a hotspot for insertion.

In this study, we described a unique xylitol utilization phenotype in RT023 (clade 3) strains made possible by the presence of a xylitol dehydrogenase gene within a mobile genetic element. We tied xylitol utilization ability to fitness advantages *in vitro*. Investigating how xylitol utilization impacts RT023 physiology and toxin production could shed light on how RT023 strains exhibit hypervirulent disease.

## MATERIALS AND METHODS

### General bacteriology

All strains used in this paper were grown as follows: glycerol stocks were streaked onto BHIS and/or BHIS + thiamphenicol (10 µg/mL) in the anaerobic chamber (5% CO_2_, 10% H_2_, and 85% N_2_) and allowed to incubate at 37°C overnight. The following evening, several colonies from the plate were inoculated into BHIS and/or BHIS + thiamphenicol and grown overnight at 37°C. Five percent of the overnight culture was inoculated into fresh medium for growth the next morning. To assess culture density, 1 mL aliquots were removed from the anaerobic chamber and measured on a spectrophotometer (OD_600_).

### Medium recipes

#### BHIS (per liter)

Thirty-seven grams of brain-heart infusion powder were dissolved per liter in deionized water, with 0.001% hemin (dissolved in 1 N NaOH and filter sterilized) added. . The medium was sterilized for 20 min at 121°C. After cooling, it was supplemented with 0.0001% vitamin K3 (dissolved in 100% EtOH and filter sterilized) and 0.03% l-cysteine.

#### BHIS (TCK)

BHIS base was supplemented with 10 µg/mL thiamphenicol, 8 µg/mL cefoxitin, and 50 µg/mL kanamycin.

#### BHIS + thiamphenicol

BHIS base was supplemented with 10 µg/mL thiamphenicol. For batch growth of the complementation strain (Δ*xdh*, *PaTc-xdh-cat*), thiamphenicol was used at a concentration of 5 µg/mL.

#### Chemically defined minimal medium (CDMM)

Amino acids, minerals, and stock solutions were added to 1 L of deionized water, filter sterilized, covered from light, and refrigerated at 4°C until used.Amino acids were added at the following concentrations: 75 mg/L each of histidine, tryptophan, and glycine; 100 mg/L tyrosine; 150 mg/L each of arginine, methionine, and threonine; 200 mg/L each of phenylalanine and alanine; 225 mg/L each of valine and isoleucine; 300 mg/L each of lysine, serine, aspartic acid, and leucine; 400 mg/L cysteine; 450 mg/L proline; and 900 mg/L glutamic acid. Minerals were added at the following concentrations: 400 mg/L KH_2_PO_4_, 1.5 g/L Na_2_HPO_4_, 900 mg/L NaCl, 5 g/L NaHCO_3_, 40 mg/L (NH_4_)_2_SO_4_, 26 mg/L CaCl_2_·2H_2_O, 20 mg/L MgCl_2_·6H_2_O, 10 mg/L MnCl_2_·4H_2_O, 1 mg/L CoCl_2_·6H_2_O, and 4 mg/L FeSO_4_·7H_2_O. Vitamins were added at the following concentrations: 0.005 mg/L vitamin B_12_, 0.125 mg/L biotin, 1 mg/L calcium d-pantothenate, 0.0125 mg/L folic acid, 1 mg/L nicotinamide, 0.05 mg/L p-aminobenzoic acid, 1 mg/L pyridoxine, 1 mg/L riboflavin, and 1 mg/L thiamine.

#### Anhydrotetracycline (aTc)

Anhydrotetracycline was prepared from a 1 mg/mL stock solution and was used at a final concentration of 100 ng/mL.

#### BRM3 base (per liter)

To deionized water, 1 g tryptone, 2 g proteose peptone #3, 2 g yeast extract, 0.4 g sodium chloride, 0.5 g bovine bile, 0.0005% hemin solution, 0.001% MgSO_4_, 0.001% CaCl_2_, 0.2% Tween 80 were added. The medium was sterilized for 40 min at 121°C. After cooling, 50 mL BRM3 supplement was added.

#### BRM3 supplement (per liter)

To deionized water, 2 g arabinogalactan, 3 g d-cellobiose, 3 g maltose, 0.8 g d-glucose, 4 g inulin, 40 g sodium bicarbonate, 0.002% vitamin K3, 0.08% K_2_HPO_4_, 0.08% KH_2_PO_4_ were added. Approximately 21 mL of concentrated HCl was added with continuously stirring. The supplement was filter sterilized and kept protected from light at 4°C until used.

#### TCCFA base (per liter)

Forty grams of proteose peptone no. 2, 5.75 g sodium phosphate monobasic monohydrate (Na_2_HPO_4_·H_2_O), 2 g potassium phosphate dibasic (KH_2_PO_4_), 2 g sodium chloride, 0.2 g magnesium sulfate heptahydrate (MgSO_4_·7H_2_O), 6 g d-fructose, and 15 g agar were added to deionized water. The medium was sterilized for 20 min at 121°C. After cooling, 20 mL of drug supplement was added.

#### TCCFA drug supplement (20 mL)

One gram of taurocholic acid (sodium salt), 250 mg d-cycloserine, and 15 mg cefoxitin were dissolved in deionized water and filter sterilized. The supplement was added to the cooled TCCFA base, and plates were poured.

#### Xylitol

A 20% (wt/vol) xylitol solution was prepared in deionized water and filter sterilized. The solution was used to supplement media with 0.5% (vol/vol).

### Biolog strain growth

Streaked strains were streaked from glycerol stocks on BHIS or BHIS in anaerobic chamber, incubated overnight at 37°C. The following evening, inoculated 5 mL BHIS with a few colonies from plates and incubated at 37°C. In the morning, inoculated 1 mL of the overnight into 5 mL fresh BHIS and grew for 4–5 h. Next, diluted subculture was then diluted 1:100 in CDMM. PM1 and PM2A Biolog plates were seeded with 100 µL per well using a multichannel pipette and covered with clear film. Optical density at 600 nm (OD_600_) was measured every 30 min for 16 h in a plate reader within an anaerobic chamber with 30 s of shaking before each measurement. Replicates from PM1 and PM2A Biolog plates were analyzed using the AMiGA (Automated Analysis of Microbial Growth Assays) software (https://github.com/firasmidani/amiga/). Area under the curve (AUC) was normalized by subtracting the no carbon (control) OD_600_. Biological replicate AUCs were then averaged. Normalized AUC values were log-transformed and a heatmap was created using R (version 4.3.1).

### Growth curves in batch

Strains were streaked from glycerol stocks on BHIS or BHIS + thiamphenicol (10 µg/mL) in an anaerobic chamber, incubated overnight at 37°C. On the following evening, 5 mL of BHIS was inoculated (with thiamphenicol when necessary) with a few colonies from plates and incubated at 37°C. The next morning, the overnight culture was inoculated into 10 mL of desired reduced media in 15 mL conical tubes to reach a starting optical density of 0.05. The measured initial concentration of overnight culture was measured to calculate concentration of subculture. Cultures were incubated anaerobically at 37°C for 8 h. One-milliliter aliquots were removed to measure OD_600_ on a spectrophotometer.

### Deletion of *xdh* using CRISPR/Cas12a

#### CRISPR/Cas12a design to delete *xdh*

This protocol was adapted from Hong et al. (33). pDL1 containing AsCpf1 (34) was linearized using BtgZI. Homologous regions to *xdh* containing the 500 bp upstream of *xdh* and 500 bp downstream of *xdh* were synthesized as a gBlock from IDT (KJW-gBlock13) with one downstream repeat located 5′ of the homologous regions. A separate gBlock was created to include the 5′ region upstream of the first repeat and the crRNA (KJW-gBlock14). gBlock #14 was PCR amplified for 20 cycles to include flanks to the plasmid and upstream crRNA site using oKJW30 and oKJW39. gBlock #14 was PCR amplified using oKJW37 and oKJW38 for 20 cycles to include flanking sequences to pDL1 and *xdh* homologous regions that could not be synthesized. gBlocks #13 and #14 were mixed at equal molarity and PCR assembled using oKJW35 and oKJW39. The resulting fragment containing repeats, crRNA, and the *xdh* homologous region was assembled into linear pDL1 using Gibson assembly to create pLPKO1. The Gibson assembly mix was transformed into EC1000 *Escherichia coli*, genotyped using oKJW35 and oKJW39, and whole plasmid sequenced. pLPKO1 was subsequently transformed into HB101, the donor strain, to create strain KJW52.1.

#### Conjugation of *C. difficile*

*E. coli* HB101 containing pLPKO1 (KJW52.1) was streaked onto BHIS with 100 µg/mL ampicillin and 20 µg/mL chloramphenicol from a glycerol stock and incubated aerobically at 37°C overnight. *C. difficile* PRB1128 recipient was streaked onto BHIS from glycerol stocks and incubated anaerobically at 37°C overnight. On the following evening, 5 mL BHIS + antibiotic (where applicable) liquid cultures were inoculated with with *E. coli* and *C. difficile*. The next morning, 1-mL aliquots were prepared of HB101 according to the number of desired conjugations. The cells were pelleted by centrifugation at 4,000 × *g* for 5 min, the supernatant was removed, and the pellets were resuspended in fresh BHIS. This step was repeated three times to remove residual antibiotics. *E. coli* pellets were transferred to the anaerobic chamber and resuspended in 200 µL of *C. difficile* culture. Aliquots (100 µL) of the mating mixture were spotted onto BHIS agar plates and incubated overnight. The next day, spots were resuspended in 2 mL BHIS, and 100 µL was spread onto BHIS(TCK) plates. The plates were incubated anaerobically at 37°C for 2–4 days. When colonies arose, restreaked onto BHIS + thiamphenicol and genotyped. Inoculated correct colonies into BHIS + thiamphenicol and grew at 37°C overnight to make 15% (vol/vol) glycerol stocks the next day.

#### Inducing CRISPR/Cas12a plasmid conjugated into PRB1128

Once the PRB1128 transconjugants grew on BHIS(TCC), they were restreaked on BHIS + thiamphenicol with 500 ng/mL anhydrotetracycline to induce the plasmid. Once the colonies grew on this plate, they were genotyped by colony PCR to verify that the genomic locus is deleted using oKJW12 + oKJW14. The deletions were confirmed using Sanger sequencing and subsequent whole-genome sequencing. To cure the plasmid, colonies were passaged 10 times over 5 days by inoculating individual colonies into 2 mL BHIS and re-inoculating 2 mL fresh BHIS with 200 µL of previous culture. On the final passage, cultures were streaked on BHIS only and BHIS + thiamphenicol. Colonies with cured plasmids were confirmed by lack of growth on BHIS + thiamphenicol. Fifteen percent (vol/vol) glycerol stocks from overnights were made from confirmed colonies grown in BHIS.

### Complementation of ∆*xdh*

#### Adapting pRPF185 to complement ∆*xdh* with titration of anhydrotetracycline

The pRPF185 vector containing a chloramphenicol acetyltransferase gene and an anhydrotetracycline-inducible promoter was used. The vector was linearized in two pieces using oKJW60 + oKJW61 and oKJW62 + oKJW63. The *xdh* coding sequence was amplified with flanks to the pRPF185 vector backbone using oKJW55 and oKJW56 and Gibson assembled with the two pieces of vector. The subsequent Gibson assembly was transformed into EC1000 *E. coli* and whole plasmid sequencing was performed by Plasmidsaurus. This confirmed plasmid (pKW16: *PaTc-xdh-cat*) was transformed into HB101 donor *E. coli* (strain KJW58.1) and selected on BHIS with 100 µg/mL ampicillin and 20 µg/mL chloramphenicol.

#### Conjugation of pKW16 into ∆*xdh*

KJW58.1 (HB101 with pKW16) and KJW107.2 (∆*xdh*) were grown and conjugation was carried out as described above (see “Conjugation of *C. difficile”*).

#### Selecting for positive transconjugants

Colonies that grew on BHIS(TCK) were restreaked on BHIS + thiamphenicol. Subsequent colonies were genotyped using oKJW55 and oKJW56 to amplify the *xdh* insert within pKW16. Incubated at 37°C for 2–4 days. When colonies arose, restreaked onto BHIS + thiamphenicol and genotyped with oKJW55 and oKJW56 to confirm the plasmid presence. Inoculated correct colonies into BHIS + thiamphenicol and grew at 37°C overnight to make 15% (vol/vol) glycerol stocks the next day.

#### Complementation in liquid media

Anhydrotetracycline was used to induce the plasmid expression at 100 ng/mL.

### Mini-bioreactor arrays (MBRAs)

#### *C. difficile* strain competitions

##### Strain growth, dilution, and mixing

Streaked strains on BHIS from glycerol stocks and grew overnight at 37°C anaerobically overnight. The following evening, inoculated a single colony into 5 mL BHIS and grew anaerobically overnight at 37°C. The next morning, inoculated 5 mL BRM3 with 250 µL overnight culture and grew until OD_600_ reached ~0.15–0.2. Diluted 1:100 in fresh BRM3 and mixed in equal volumes to create the competition inocula.

##### Inoculation

Septa were bleached for 10 min before inoculating 150 µL of the desired inoculum into each well containing 15 mL BRM3 using a 22G × 3 in. hypodermic needle. Negative-control wells were inoculated with sterile BRM3. Flow was initiated immediately after inoculation.

##### Determination of inoculation dose using viable plating

Individual diluted cultures and competition mixes were serially diluted in sterile 1× PBS to 10^−5^. Aliquots (100 µL) of the appropriate dilutions were spread onto BHIS agar plates and incubated anaerobically at 37°C overnight. Colonies were counted from plates containing 30–300 colonies, and the viable cell concentration was calculated as CFU/mL.

##### Flow information

BRM3/BRM3 + 0.5% xylitol was supplied at a flow rate of one inflow and two outflow channels using peristaltic pumps, to achieve an 8-h turnover.

### Sampling over time

Inoculum cultures were saved (day 0) for genomic DNA extraction. For six consecutive days, 500 µL samples from each reactor were collected in a microfuge tube. The samples were centrifuged at 4,000 × *g* for 5 min to pellet the cells, and the resulting pellets were stored at −20°C until further processing.

#### Strain growth, dilution, and inoculation

Strains were streaked on BHIS from glycerol stocks and incubated anaerobically at 37°C overnight. The following evening, a single colony was inoculated into 5 mL BHIS and incubated anaerobically overnight at 37°C. The next morning, 5 mL BRM3 was inoculated with 250 µL of the overnight culture and incubated until OD_600_ reached ~0.15–0.2. The culture was then diluted 1:100 in fresh BRM3 to prepare the inoculum. Septa were bleached for 10 min before inoculating 150 µL of the desired inoculum into each well using a 22G × 3 in. hypodermic needle. Negative-control wells were inoculated with sterile BRM3.

#### Flow information

BRM3/BRM3 + 0.5% xylitol was supplied at a flowrate of one inflow and two outflow channels using peristaltic pumps, to achieve an 8-h turnover.

#### Sampling over time

Inocula cultures were saved (day 0) for genomic DNA extraction. For six consecutive days, 500 µL samples from each reactor were collected. The samples were centrifuged at 4,000 × *g* for 5 min to pellet the cells, and the resulting pellets were stored at −20°C until further processing.

### *C. difficile* invasion of a fecal community

#### Establishing a fecal community in MBRAs

This protocol was adapted from Auchtung et al. (29). One 12 mL fecal stock (CCHF008) was rapidly defrosted in a 37°C water bath and was grown in 20 mL of reduced BRM3 anaerobically overnight at 37°C. The following morning, 1 mL was inoculated into each reactor, and the in/out flow was turned on at a rate of 8 h (one in, two out on peristaltic pumps). Twenty-four hours later, each reactor was individually dosed with 25 mg/mL clindamycin and fed BRM3 + clindamycin or BRM3 + 0.5% xylitol + clindamycin in medium bottles for 4 days. No clindamycin control strips were included. On the fourth day (day −1), the clindamycin treatment was stopped by switching to BRM3 + 0.5% xylitol or BRM3 only bottles. Twenty-four hours later, *C. difficile* was inoculated.

#### *C. difficile* growth and inoculation into MRBAs

Growth was performed the same way as described above (“Strain growth, dilution, and inoculation”). For this experiment, *C. difficile* was diluted 1/10 in BRM3 once it reached the desired optical density. One hundred fifty microliters of dilution was inoculated into each respective well.

#### Enumeration of *C. difficile* by selective plating

One-milliliter samples were collected each day, and 100 µL was used to serially diluted to 10^−3^ in reduced phosphate buffered saline. Five microliters of each dilution series was drip-plated on BHIS + TCCFA (see above for a detailed recipe) by tilting the plate 30° and allowing the dilution to run down the plate. The following day, visible colonies were counted, and CFU/mL was quantified.

### Genomic DNA extraction

Genomic DNA was extracted from 1 mL pellets using the DNeasy 96 PowerSoil Kit (Qiagen). DNA was eluted in a final volume of 60 µL, and DNA concentration was determined using 2 µL in the Quant-iT high-sensitivity dsDNA protocol (Invitrogen). A standard curve was generated each time using the standards provided by the kit.

### qPCR to distinguish strains in competition

Genomic DNA from all samples was normalized to 1 ng/µL in nuclease-free water. Quantitative PCR (qPCR) was performed in 10 µL reaction volumes containing 5 µL of 2× SYBR Green master mix, 3 µL nuclease-free water, 1 µL genomic DNA, and 1 µL of a mixed forward and reverse primer solution (10 µM each). Technical duplicates were performed for each sample. For competitions between PRB1128 and CD2015, primers oKJW32 + oKJW33 (*thyX*) and oKJW84 + oKJW85 (*thyA*) were used, respectively. For competitions between Δ*xdh* (PRB1128) and CD2015, primers oKJW32 + oKJW33 (*thyX*) and oKJW84 + oKJW85 (*thyA*) were used, respectively. For competitions between PRB1128 and Δ*xdh* (PRB1128), primers oKJW32 + oKJW33 and oKJW15 + oKJW16 (*xdh*) were used, respectively. Reactions were cycled in an Applied Biosystems QuantStudio qPCR system for 40 cycles using ∆∆Ct method in a 10 µL volume.

### Generation of competitive index

From the raw data files, we calculated the average of the technical replicate Ct values. We calculated the ratios at days 0–6 using the 2^−∆∆Ct^ method: 2^−((Ct(*thyX*)−Ct(*thyA or xdh*))^. Specifically, we determined the ratio of each strain by calculating 2^−(*thyX−thyA*)^ on each day, including day 0. To calculate the competitive index, we divided the ratio of strains on the respective day by the ratio of strains on day 0 (inoculum). The competitive index was log_10_-transformed, and the medians were plotted according to the day of competition. *thyX* is intact in RT023s, while RT027s have four genes inserted at the *thyX* locus (including *thyA*). Therefore, *thyX* amplification demonstrates the abundance of RT023s, and *thyA* demonstrates the abundance of RT027s.

### Alignment and phylogenetic analysis of a genomic hotspot across clades for gene insertions

Nucleotide BLAST of the intact HTH transcriptional regulator revealed MGE insertions in clades 1 and 4 in addition to clade 3. Genomic neighborhoods containing the 5′ and 3′ pieces of the HTH, encompassing the inserted genes, were aligned using MEGA12 Aligner (35). A phylogeny was made using the Tree Explorer plugin and was inferred using the Maximum Likelihood method and the Tamura-Nei 1993 model (36, 37).

### Structural modeling

The XYL2 homology model was acquired from the *Saccharomyces* genome database (modeled using AlphaFold based on XYL2 from *Saccharomyces cerevisiae* S288C). The amino acid sequence of Xdh from DSM102859 was used to generate a homology model using AlphaFold version 2.3.1. The highest confidence models were aligned (using “align” function, which calculates an RMSD value) in PyMOL version 2.5.5. The alignment figure was also created in PyMOL.

### 16S rRNA sequencing of a fecal community

One-milliliter -samples were collected at day 6 of *C. difficile* challenge and stored at −20°C until genomic DNA extraction was performed. See above for genomic DNA extraction details. Purified DNA was sent for V3–V4 16S rRNA sequencing to SeqCoast Genomics with 50k reads, 2 × 300 bp paired ends. Genera were identified using Qiime2-2023.9. Reads were filtered in Qiime using the quality-filter command according to *q*-score. Deblur was used to trim 250 bp from the reads. The V3 and V4 regions were considered in the Silva classifier to assign taxonomy. A Biological Observation Matrix (BIOM) file was created with OTU information, and relative abundances were visualized using Agile Toolkit for Incisive Microbial Analyses (Baylor College of Medicine).

## Data Availability

Data that support these findings, figures, and supplemental materials are available through figshare using DOI: 10.6084/m9.figshare.c.8101924.

## SUPPLEMENTAL MATERIAL

Supplemental material msphere.00263-26-s0001.pdf Fig. S1-S3 and Tables S1-S5.

ASM does not own the copyrights to Supplemental Material that may be linked to, or accessed through, an article. The authors have granted ASM a non-exclusive, world-wide license to publish the Supplemental Material files. Please contact the corresponding author directly for reuse.
