# Supplementary material for: Acquisition of a gene cluster in Clostridioides difficile PCR ribotype 023 strains enables xylitol utilization
Source: mSphere. 2026 Jul 16;11(8):e00263-26. doi: 10.1128/msphere.00263-26 (PMC13508786; doi:10.1128/msphere.00263-26)
Supplement: Supplementary file 1 [file msphere.00263-26-s0001.pdf]

SUPPORTING INFORMATION FOR:

Acquisition of a gene cluster in *Clostridioides difficile* PCR ribotype 023 strains enables xylitol utilization

Katherine J. Wozniak[1], Lei Pan[1], Duolong Zhu[1], Jeroen Corver[2], Ed J. Kuijper[2], Wiep Klaas Smits[2], Robert A. Britton[1,3#]

[1]Department of Molecular Virology & Microbiology, Baylor College of Medicine, Houston, TX, USA
[2]Leiden University Center for Infectious Diseases (LUCID), Leiden Medical Center, Leiden, The Netherlands
[3]Alkek Center for Metagenomics & Microbiome Research, Baylor College of Medicine, Houston, TX, USA

This PDF file includes:

Figures S1 to S3
Legends for Figures S1 to S3
Tables S1 to S5
References

FIGURES

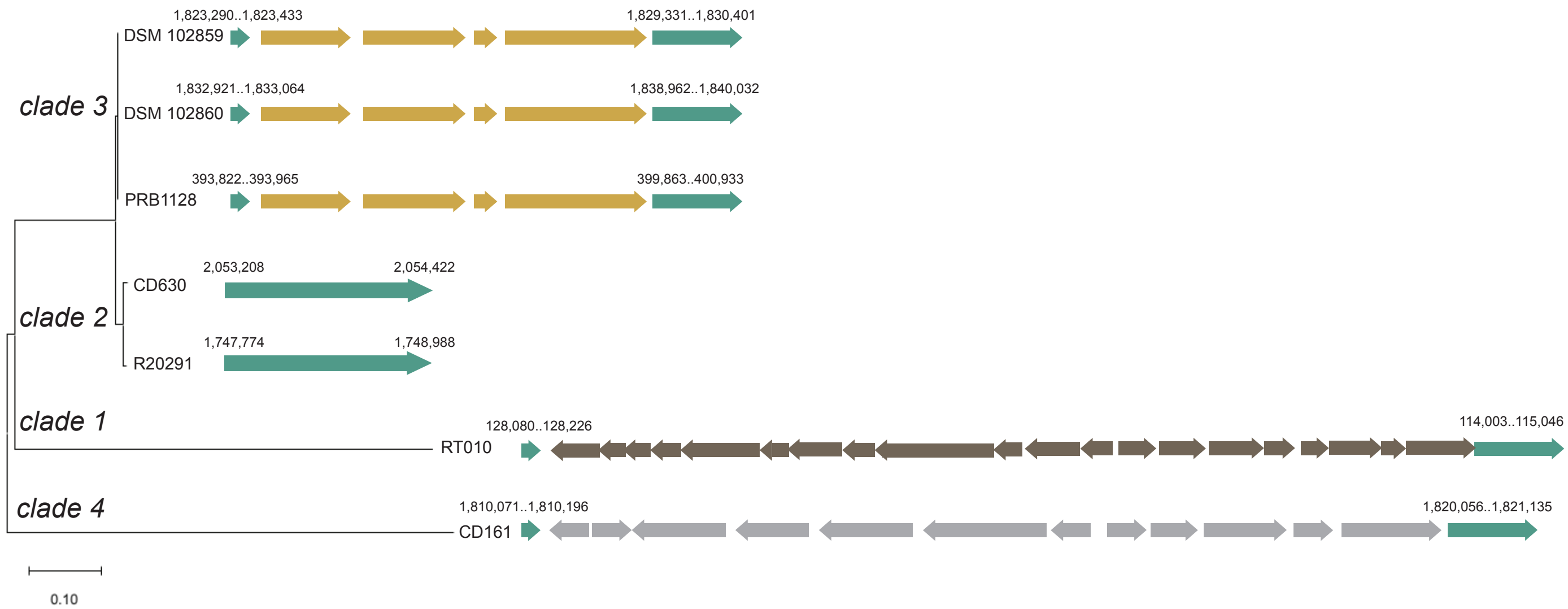

Supplemental Figure 1. Neighborhood diagrams of MGE insertion sites. Phylogeny of representative strains from clades 1-4 organized according to relatedness of the intact HTH transcriptional regulator, revealing this site is a hotspot for MGE insertion. Clade 3 strains DSM 102859 (Genbank: CP020378.1) and DSM 102860 (Genbank: CP020379.1) have identical insertions to PRB1128 (this study). Genes in teal are the 5' and 3' HTH transcriptional regulator. Clade 2 strains CD630 (Genbank: AM180355.1) and R20291 (Genbank: CP115183.1) encode an intact transcriptional regulator (teal). RT010 (Genbank: CP118392.1) encodes a recombinase, hypothetical, relaxase, replication initiator, VLP-containing, ATPase, transcriptional regulator, N-acetyltransferase, topoisomerase (partial), peptidase, *ermB*, peptidase, topoisomerase (partial), *parA*, peptide-binding, antitoxin, zeta family toxin, and ATPase. CD161 (Genbank: CP029154.1) contains a hypothetical, hypothetical, *aadE*, N-acetyltransferase, transcriptional regulator, bifunctional aminoglycoside N-acetyltransferase AAC(6'), N-acetyltransferase, conjugal transfer, *repA*, plasmid recombinase, hypothetical, and DUF4368 containing gene. Genes in teal are the 5' and 3' HTH transcriptional regulator, while genes in brown or grey are unique to their respective clade. Numbers indicate genomic position within the HTH transcriptional regulator that the MGE inserted (if applicable).

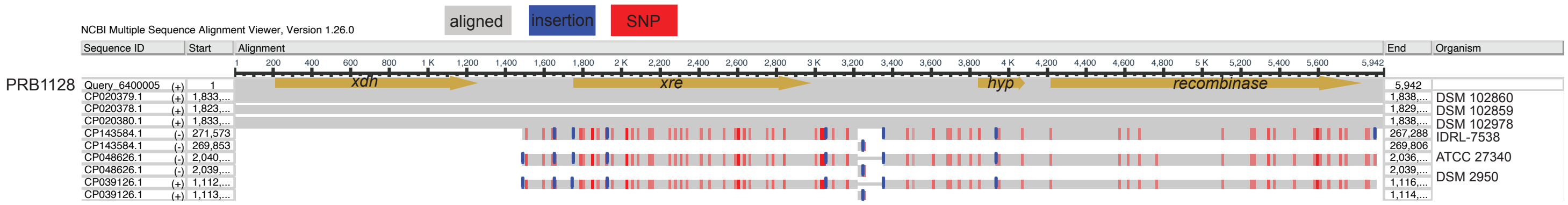

Supplemental Figure 2. Nucleotide BLAST results of MGE in PRB1128. PRB1128 MGE was used as the query to find aligned regions in the NCBI database. Sequence ID indicates the genome accession number for *C. difficile, E. faecalis,* and *B. producta.* Grey regions indicate alignment, grey dashes indicate gaps, blue lines indicate insertions, and red lines indicate SNPs.

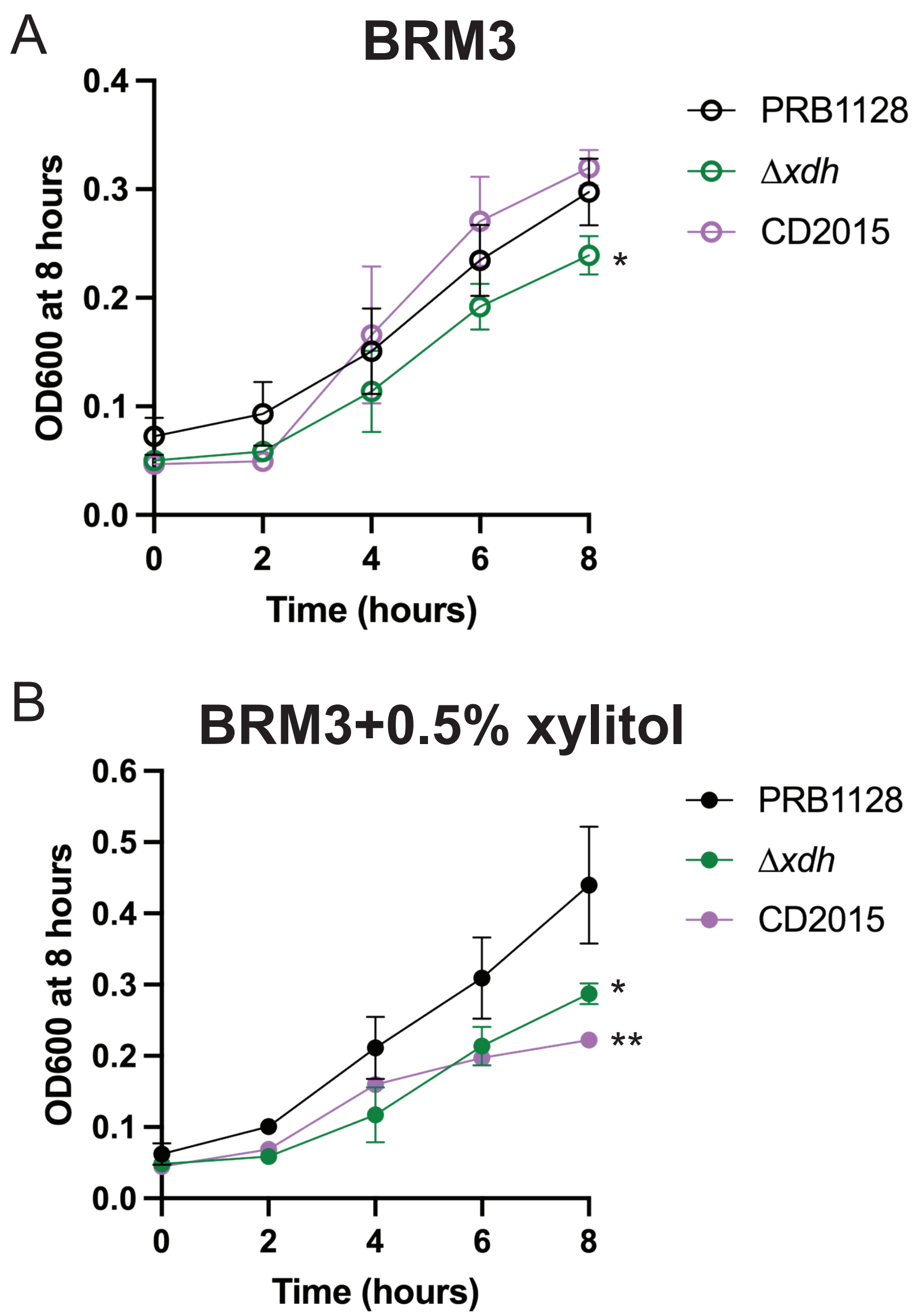

Supplemental Figure 3. Growth curves in BRM3 and BRM3+xylitol. A. PRB1128, Δ*xdh*, and CD2015 grown in BRM3 medium for 8 hours. Mean and error shown. n=3-4 biological replicates per group. Optical density of Δ*xdh* is significantly different from PRB1128 at 8 hours in BRM3 (*$p<0.01$). **B.** PRB1128, Δ*xdh*, and CD2015 grown in BRM3 with 0.5% xylitol for 8 hours. Mean and standard deviation shown. n=4 biological replicates per group. Optical density of Δ*xdh* and CD2015 are significantly different from PRB1128 at 8 hours in xylitol (*$p<0.01$, **$p<0.005$).

Supplemental Table 1. Investigation of MGE origin in RT023s. Top nucleotide BLAST results of the XRE transcriptional regulator, hypothetical, and recombinase. Query cover indicates the percentage of sequence matching to the database. Unique accession numbers to top hits listed to identify specific genomes that the alignments originated from.

Gene	Scientific Name	Query Cover	E-value	% identity	Accession
xre transcriptional regulator	*Enterococcus faecalis*	100	0	93.25	LR962119.1
	Enterococcus faecalis	100	0	92.84	LR536670.1
	Enterococcus faecalis	100	0	92.84	LR135488.1
	Enterococcus faecalis	100	0	92.84	LR962608.1
	Enterococcus faecalis	100	0	92.84	LR961970.1
	Enterococcus faecalis	100	0	92.84	LR961953.1
	Enterococcus faecalis	100	0	92.84	LR961925.1
	Enterococcus faecium	100	0	92.76	CP041261.3
	Enterococcus faecium	100	0	92.76	CP041270.2
hypothetical	*Blautia producta*	100	1.00E-89	95.73	CP048626.1
	Blautia producta	100	2.00E-88	95.73	CP039126.1
	Blautia hansenii	100	8.00E-87	95.24	CP022413.2
	Blautia hansenii	100	8.00E-87	95.24	CP102264.1
	Enterococcus faecium	100	8.00E-87	95.24	AP026593.1
	Enterococcus faecium	100	8.00E-87	95.24	AP026586.1
	Enterococcus faecium	100	8.00E-87	95.24	AP026579.1
recombinase	*Blautia producta*	99	0	94.91	CP048626.1
	Blautia producta	99	0	94.91	CP039126.1
	Enterococcus faecalis	100	0	94.18	LR962119.1
	Enterococcus faecalis	100	0	94.11	LR536670.1
	Enterococcus faecalis	100	0	94.11	LR135488.1
	Enterococcus faecalis	100	0	94.11	LR962836.1
	Enterococcus faecalis	100	0	94.11	LR962098.1
	Enterococcus faecalis	100	0	94.11	LR961970.1
	Enterococcus faecalis	100	0	94.11	LR961953.1
	Enterococcus faecalis	100	0	94.11	LR961925.1

Supplemental Table 2. Results of Tukey's multiple comparisons tests on growth data.

Medium	Comparison	Adj. p-value	Significant?
CDMM	PRB1128 vs. Δ*xdh*	0.1897	n.s.
	PRB1128 vs. Δ*xdh, PaTc-xdh-cat*	0.0256	*
	PRB1128 vs. Δ*xdh, PaTc-xdh-cat*+aTc	0.9975	n.s.
	Δ*xdh* vs. Δ*xdh, PaTc-xdh-cat*	0.8173	n.s.
	Δ*xdh* vs. Δ*xdh, PaTc-xdh-cat* +aTc	0.2962	n.s.
	Δ*xdh, PaTc-xdh-cat* vs. Δ*xdh, PaTc-xdh-cat* +aTc	0.0523	n.s.
CDMM+xylitol	PRB1128 vs. Δ*xdh*	0.0098	**
	PRB1128 vs. Δ*xdh, PaTc-xdh-cat*	0.0396	*
	PRB1128 vs. Δ*xdh, PaTc-xdh-cat*+aTc	0.9901	n.s.
	Δ*xdh* vs. Δ*xdh, PaTc-xdh-cat*	0.9829	n.s.
	Δ*xdh* vs. Δ*xdh, PaTc-xdh-cat* +aTc	0.0288	*
	Δ*xdh, PaTc-xdh-cat* vs. Δ*xdh, PaTc-xdh-cat* +aTc	0.0921	n.s.
CDMM vs. CDMM+xylitol	PRB1128	<0.0001	****
	Δ*xdh*	0.0075	**
	Δ*xdh, PaTc-xdh-cat*	0.0004	***
	Δ*xdh, PaTc-xdh-cat* +aTc	0.0003	***

Supplemental Table 3. Strains used in this study.

Strain genotype	Source	Strain number	Ribotype
PRB1128	This paper	KJW29.1	RT023
PRB1128, Δ*xdh*	This paper	KJW107.2	RT023
PRB1128, Δ*xdh* , *PaTc-xdh-cat*	This paper	KJW108.1	RT023
CD4011	(1)	PRB278	RT001
CD3014	(1)	PRB279	RT001
CD4004	(1)	PRB284	RT002
CD2012	(1)	PRB285	RT002
CD37	Sorg Lab, Texas A&M	PRB236	RT009
CD630	Sorg Lab, Texas A&M	PRB238	RT012
CD1014	(1)	PRB282	RT014
CD305	(2)	PRB1100	RT023
LUMCMM07-0002 (5)	This paper	PRB1110	RT023
LUMCMM07-0003 (7004810)	This paper	PRB1111	RT023
LUMCMM09-0007 (FR1406)	This paper	PRB1120	RT023
LUMCMM09-0010 (SE1109)	This paper	PRB1123	RT023
LUMCMM10-0006 (CVI65)	This paper	PRB1130	RT023
CD196	Sorg Lab, Texas A&M	PRB237	RT027
CD3017	(1)	PRB268	RT027
CD4001	(1)	PRB291	RT027
R20291	(1)	PRB468	RT027
CD2015	(1)	KJW31.1	RT027
CD1007	(1)	PRB280	RT053
CD2048	(1)	PRB281	RT053
CD2001	(1)	PRB288	RT078
CD1015	(1)	KJW32.1	RT078
VPI 10463	(3)	PRB239	RT087
CD2046	(1)	PRB289	unique
CD2018	(1)	PRB283	unique
EC1000	Lab stock	KJW2.1	*Escherichia coli*
HB101 with pRK24 for conjugation (Tra+, Mob+, AmpR, TcR)	Lab stock	KJW52.1	*Escherichia coli*
HB101 (AmpR) pRPF185-*xdh-cat*	This paper	KJW58.1	*Escherichia coli*
HB101, pWH34-*PtetM-AsCpf1-xdh gRNA-xdhHomology-cat*	This paper	KJW53.1	*Escherichia coli*

Supplemental Table 4. Primers used in this study.

Primer	Sequence (5'-3')	Description	Source
oKJW12	GCAAAGTAGAAAAGAGAGAGCCT	*xdh* genotyping	This paper
oKJW14	TGTGTTACTCCGTATTCCTCACT	*xdh* genotyping	This paper
oKJW15	TAGCGGGTTCGGCAATAAG	*xdh* qPCR	This paper
oKJW16	TCCATGACCTGTTCCTCAATAC	*xdh* qPCR	This paper
oKJW30	TAATTTCTACTCTTGTAGATAGGTTATACTTAAACTTAATTAATAGATTTTAT	extending crRNA flank	This paper
oKJW32	CCAGTTGCGACAGACGAAAT	*thyX* qPCR	This paper
oKJW33	TGAACAAGCCCTTGAAATAAC	*thyX* qPCR	This paper
oKJW35	CTTTTTTTATTAAATAAGAGTATAAAATAAAGTTAAAAGAAGAAAATA	assembling crRNA and *xdh* flanking region	This paper
oKJW37	AAGTTTAAGTATAACCTATCTACAAGAGTAGAAATTATTCATTGCAAATCCTTTCAT	Extending *xdh* flank to have pLPKO1 overlap	This paper
oKJW38	TATAAAATAAAGTTAAAAGAAGAAAATATAATTTCTACTCTTGTAGATAT	Extending *xdh* flank to have pLPKO1 overlap	This paper
oKJW39	CATGCTGATCTAGATTTCTCCATAGCTCCAATCAAAAAAGGCAACTACCA	Extending crRNA to have *xdh* overlap	This paper
oKJW55	CAGATAGGAGGGTTTGATAATGAAAGGATTTGCAATGAAGAAAATC	*xdh* insertion in pRPF185	This paper
oKJW56	GCACTCTTTAGTCTAAGATTATTATATAAAGACGATTGGTTTTATCAAAT	*xdh* insertion in pRPF185	This paper
oKJW60	AGTCTAAGATTATTATATCAAACCCTCCTATCTGAGCTCAGATCTG	pRPF185 linearization (piece 1)	This paper
oKJW61	GCAATTCTCGTACAATTATTTTACCAAAAGAAATTTTTATTAATTAG	pRPF185 linearization (piece 1)	This paper
oKJW62	CAGATCTGAGCTCAGATAGGAGGGTTTGATATAATAATCTTAGACT	pRPF185 linearization (piece 2)	This paper
oKJW62	CTAATTAATAAAAATTTCTTTTGGTAAAATAATTGTACGAGAATTGC	pRPF185 linearization (piece 2)	This paper
oKJW84	GATGGCCAGCCTGCTCATACAA	*thyA* qPCR	This paper
oKJW85	TGTTTCATCAGCCCAGCTATCCCA	*thyA* qPCR	This paper

Supplemental Table 5. Plasmids used in this study.

Plasmid name	Genotype	Source	Antibiotic cassette
pLPKO1	pDL1-*PtetM-AsCpf1-xdh_gRNA-xdhHomology-cat*	This study	chloramphenicol
pKW16	pRPF185-P*aTc-xdh-cat*	This study	chloramphenicol

REFERENCES

1. Physiological and ecological investigations of *Clostridium difficile* - ProQuest. https://www.proquest.com/openview/a0631d9220ae0a4bd2ada5d0a89fa04d/1?pq-origsite=gscholar&cbl=18750.
2. Shaw, H. A. *et al.* Clostridium difficile clade 3 (RT023) have a modified cell surface and contain a large transposable island with novel cargo. *Sci Rep* **9**, 15330 (2019).
3. Wehrhahn, M. C. *et al.* A series of three cases of severe *Clostridium difficile* infection in Australia associated with a binary toxin producing clade 2 ribotype 251 strain. *Anaerobe* **55**, 117–123 (2019).
